# Effects of Lycium barbarum Polysaccharides on Health and Aging of *C. elegans* Depend on *daf-12/daf-16*

**DOI:** 10.1155/2019/6379493

**Published:** 2019-09-10

**Authors:** Zhaokang Zhang, Yannan Zhou, Haitao Fan, Kirunda John Billy, Yunjie Zhao, Xuan Zhan, Lijian Yang, Ya Jia

**Affiliations:** ^1^Institute of Biophysics and Department of Physics, Central China Normal University, Wuhan 430079, China; ^2^College of Bioengineering, Beijing Polytechnic, Beijing 100029, China

## Abstract

As the global population ages, searching for drugs and functional foods which can slow down the aging process has attracted a number of researchers. In this paper, the Lycium barbarum polysaccharides (LBP) extracted from Lycium barbarum was characterized and the effects of LBP on the aging and health of *C. elegans* were studied. Results showed that LBP can prolong the lifespan, improve the abilities to withstand environmental stress, enhance reproductive potentials, and maintain muscle integrity of *C. elegans*. By using genetically mutated *C. elegans* strains, RNAi gene silencing, and measuring the mRNA expression level, it was demonstrated that the lifespan of *C. elegans* was extended by LBP mainly through *sir-2.1*, *daf-12*, and *daf-16*. The present study might provide a basis for further study of LBP as a food or drug to interfere with aging and reduce the incidence of age-related diseases.

## 1. Introduction

Aging is defined by the time-dependent functional decline of living organisms, during which the self-renewal and repair abilities of the organism are weakened [1]. Aging is often accompanied by a gradual decline in environmental adaptability, deterioration of physiological functions, and increase of vulnerability to diseases (such as hardening of the arteries, cancer, and Alzheimer's disease) [1, 2]. As the global population ages, searching for drugs, which can treat age-related diseases, slow down the normal aging process, and prolong lifespan, is an important aspect of current aging researches [3–5]. In addition, genetic pathways that regulate the lifespan have been shown to be evolutionarily conserved; therefore, revealing the molecular mechanism of using drugs to extend the lifespan of *C. elegans* can help to better understand the biological mechanisms related to lifespan [6].

As people pay more attention to the quality of life, it is more and more popular to choose drugs and functional foods which may reduce the risk of death. Therefore, a traditional Chinese herb, Lycium barbarum, has become the focus of many studies [7]. Lycium barbarum is widely grown in the western and northern regions of China (such as Gansu province, Ningxia province) [8]. It is generally used to improve vision, nourish the liver and kidneys, and delay aging [9, 10]. Due to its excellent health care function, it has been very popular not only in Asia but also in other continents [11, 12]. Recent medical research has shown that Lycium barbarum contains a variety of nutrients, with Lycium barbarum polysaccharides (LBP) as one of its main active ingredients [13].

The LBP can usually be obtained by water extraction and alcohol precipitation, ultrasonic assistance, or enzymatic hydrolysis [14]. It was reported that the main pharmacological effects of LBP include regulating immunity [15, 16], blood sugar, and lipids [17]; preventing tumors [18, 19]; and delaying aging [20–25]. By analyzing the expression levels of interleukin-2 and tumor necrosis factor-alpha-related mRNA and protein in human peripheral blood mononuclear cells, Gan et al. [15] showed that LBP can increase the activity of interleukin-2 and tumor necrosis factor-alpha, which can induce the immune responses. Chen et al. [16] revealed that LBP can enhance the ability of dendritic cells to respond to Th1 and Th2 and then enhance hosts' immunity. By feeding mice with food of different fat contents and LBP, Ma et al. [17] found that LBP has no significant effect on the body weight of the mouse but significantly reduces blood lipids, lowers blood glucose, and inhibits lipid oxidation. Gan et al. [18] reported that feeding LBP to the mice inoculated with sarcoma S180 cells can inhibit the growth of the sarcoma S180, increase the immunity, improve the phagocytic function of macrophages, and proliferate spleen lymphocytes; thus, LBP can prevent tumors in the mouse. Luo et al. [19] demonstrated that LBP can induce apoptosis of prostate cancer cells (PC-3 and DU-145) in in vitro cell culture and inhibit the growth of prostate cancer in the nude mouse xenograft tumor model.

Up to now, the investigations on the effects of LBP on the aging process have been focused on eliminating free radicals and delaying skin aging [20–25]. In various antioxidant systems in vitro, Li et al. [20] indicated that LBP has the function of antioxidant remedies, such as inhibition of 1,1,2-diphenyl-2-picrylhydrazyl (DPPH) free radicals, superoxide scavenging ability, hydrogen peroxide-mediated erythrocyte hemolysis, and ferrous ion sequestration in mice. Lin et al. [21] showed that LBP can effectively scavenge DPPH and ABTS+free radicals, superoxide anion, and hydroxyl radical. Tian et al. [22] reported that LBP can enhance the antioxidation capacity of chicken embryo liver cells, via resisting the decrease of the activity of chicken embryo liver cells induced by H_2_O_2_, reducing the ROS content and promoting the activity of antioxidant enzymes. Liang and Zhang [23] proved that LBP can delay skin aging through significantly increasing skin water content, skin thickness, subcutaneous thickness, and fibroblast count of the mice and improving the tissue of decayed skin. However, there is little direct evidence that the LBP can extend lifespan.

In this paper, the LBP used was extracted from Ningxia Lycium barbarum. First, we characterized the molecular weight distribution, monosaccharide composition, and infrared absorption spectrum of LBP. Then, the effects of LBP on lifespan and health of *C. elegans* were investigated and it was shown that LBP extends the lifespan, improves the abilities to withstand environmental stress, boosts reproductive potentials, and maintains muscle integrity. Finally, by using genetic mutated *C. elegans* strains, RNAi gene silencing, and examining mRNA expression level, our results revealed that the LBP extended lifespan mainly through *sir-2.1*, *daf-12*, and *daf-16*.

## 2. Materials and Methods

### 2.1. Preparation of LBP

The LBP from Ningxia Lycium barbarum was prepared by water extraction, alcohol precipitation, deproteinization, and recrystallization. In brief, the fruits of Ningxia Lycium barbarum were vacuum dried in a vacuum drying oven at 60°C, taken out, and placed in a desiccator for 24 h. We took 10 g of the crushed element and placed it in a normal reflux device, then added 20 ml chloroform+methanol in the ratio of 2 : 1 each time. We degreased twice at 60°C. Filtration was done, the liquid was discarded, and the residue was vacuum dried. Then, 20 ml of 80% ethanol was added and the mixture was refluxed twice at 60°C to recover ethanol. After that, the mixture was extracted twice with water at 60°C, with the liquid-solid ratio being 1 : 20. The resultant compound was concentrated, precipitated 4 times with 95% ethanol, and kept in the oven for 24 hours. Filtration was then done by suction. The product was washed with 95% ethanol, absolute ethanol, and acetone, then vacuum dried (50°C) to obtain a crude powder of LBP. Finally, the product was deproteinized and recrystallized.

### 2.2. Characterization of LBP

#### 2.2.1. Chemical Composition Analysis

We measured the carbohydrate content of LBP by phenol-sulfuric acid method [26], determined the content of uronic acid by m-hydroxydiphenyl method [27], and then detected the protein content by BCA method. The retention time (RT) of the dextran standard was determined by HPLC-GPC, and a standard curve was drawn. The HPLC-GPC was then used to detect the molecular weight distribution of the LBP.

#### 2.2.2. Determination of the Composition of LBP Monosaccharides

5 mg of LBP was dissolved in 2 mol/L TFA, hydrolyzed at 99°C for 5 h, removed with acid by rotary distillation, and then added 0.5 ml of 4% sodium borohydride solution. The resultant solution was placed at room temperature for 1.5 h and added acetic acid dropwise until no bubbles were generated, and then, the concentration process was repeated. Next, the concentrated sample was vacuum dried, 1 ml of pyridine and n-propylamine was added, and the mixture was placed in a water bath at 55°C for 30 min. The mixture was vacuum dried, 0.5 ml of pyridine and acetic anhydride was added followed by heating at 95°C for 1 h, blown dried with nitrogen, and vacuum dried. The mixture was then dissolved in chloroform for GC-MS analysis.

#### 2.2.3. Determination of FT-IR Spectra

The LBP was mixed with potassium bromide powder and pressed into millimeter-sized sheets, and the FT-IR spectrum of LBP was recorded by using the Fourier transform infrared spectrometer in the frequency range of 4000-500 cm^−1^.

### 2.3. Strains and Culture

*C. elegans* strains (N2 (wild type), BA17 (fem-1(hc17) IV.), VC199 (sir-2.1(ok434) IV.), GR1307 (daf-16(mgDf50) I.), CF1038 (daf-16(mu86) I.), and DR1407 (daf-12(m583) X.)) were obtained from the Caenorhabditis Genetics Center which is supported by the National Institutes of Health and from Professor Huairong Luo, Southwest Medical University. *E. coli* OP50 was provided by Professor Zhengxing Wu, Huazhong University of Science and Technology. The other bacterial strains were obtained from Professor Ge Shan, University of Science and Technology of China, and from Professor Huairong Luo. Strains were kept at 25°C according to standard methods, cultured on NGM plates, and seeded with *E. coli* OP50 [28].

### 2.4. Lifespan Analysis

The worms synchronized to the L4 stage were picked and provided with enough *E. coli* OP50 as food on ordinary NGM plates with different concentrations of LBP. These NGM plates had 25 *μ*M 5-fluoro-2′-deoxyuridine (FUdR), and we replaced any NGM plate when eggs were found during the experimental process. Worms were observed every 24 hours until all of them were dead. Worms were scored dead if they did not move when gently touched by the worm picker. Those that disappeared from the plate and died prematurely from internal hatching or vulval rapture were excluded from the analysis.

For lifespan experiments involving RNAi, HT115 bacteria containing an empty vector L4440 or RNAi plasmid were used instead of *E. coli* OP50 on NGM plates (these NGM plates contained carbenicillin (25 mg/ml) and IPTG (1 mM), excluding FUdR) and the other operations were the same as mentioned above. The bacteria for RNAi were from the Ahringer library.

### 2.5. Lifespan under Different Stressors

Worm culture for the following assays was the same as in the previous ordinary lifespan assay. After 5 days, respective stress analyses were done as follows: (a) For the heat stress assay, worms were transferred to new corresponding NGM plates. Then, their survival at 37°C was monitored every 2 hours until they were all dead. (b) For the osmotic, metal, and oxidative stress analysis, worms were transferred to S-buffer solutions (0.5 M KH_2_PO_4_, 0.5 M K_2_HPO_4_, and 0.1 M NaCl) containing 300 mM NaCl (for osmotic stress), 50 *μ*M CdCl_2_ (for metal stress), and 30 mM H_2_O_2_ (for oxidative stress). Then, the survival was monitored hourly until all of worms died.

### 2.6. Self-Brood Size and Egg Production Rate

After bleaching, L1 stage worms were placed on the NGM plates seeded with enough *E. coli* OP50 and with different concentrations of LBP. After worms developed to the L4 stage, they were transferred to their new corresponding NGM plate every day until the cessation of egg production. A single worm was cultured on an individual NGM plate. The number of eggs produced was determined by the progeny size.

### 2.7. Motility Measurement

Worm culturing was the same as in the ordinary lifespan assay. Motility was measured on day 1 (L4 stage), day 6, day 11, and day 16. The animal's motile ability is divided into three categories A, B, or C [29]. Worms which can spontaneously move sinusoidally are put in Class A. On stimulation, worms that are unable to move sinusoidally but can still move belong to Class B. Worms that can only move the head or the tail on stimulation are placed in Class C.

### 2.8. Determination of Antioxidant Enzyme Activity and ROS Content

Worm culturing was the same as in the ordinary lifespan assay (all the NGM plates had 150 *μ*M FUdR). On day 5, the NGM plates were washed with S-buffer and the worms were collected in an EP tube. The worms were washed 3 times with sterile physiological saline, and sterile physiological saline was added. The mixture was homogenized on ice and centrifuged, and the supernatant was taken for use. A portion of the product was mixed with a DCFDA probe solution or according to the SOD/CAT kit instructions, and the fluorescence was measured using a fluorescent plate reader. Simultaneously, the other portion of the product was used for determining protein concentration with the BCA method. The relative values of the control and test groups were determined by calculating the fluorescence values of the unit protein concentration.

### 2.9. Relative Expression of mRNA Analysis

Worms were cultured in the same way as in the ordinary lifespan assay. On day 5, the NGM plates were washed with S-buffer and the worms were collected in an EP tube. We washed the worms 3 times with S-buffer, and RNA was extracted by the rapid freeze-thaw lysis and TRIzol/chloroform method. Then, RNA was reverse transcribed into cDNA. The relative expression levels of the genes were detected using the real-time PCR according to the SYBR green Mix instructions. *act-1* gene was used as the internal reference gene. There were three setups for each gene to be tested, and the entire experiment was repeated three or more times. The primers used for PCR are shown in Table 1.

### 2.10. Statistical Analysis

All tests were repeated at least three times. Mean lifespan was defined as the average lifespan, and the error was the standard deviation from multiple results. Log-rank test was used to obtain the mean lifespan and the corresponding *P* values. Maximum lifespan was the average lifespan of 10% longest worms, and the error was the standard deviation of multiple results. The number of eggs was the average of multiple outcomes with an error of the standard deviation from each individual. Others were multiple experimental means and standard deviations. Softwares Origin8, SPSS-22, and GraphPad Prism 8.1.2 were used for statistical analysis and plotting of the experimental data. *P* < 0.05 for significant differences, and *P* < 0.01 for extremely significant differences in hypothesis test analysis.

## 3. Results

### 3.1. Chemical Composition of LBP

**Figure 1:**
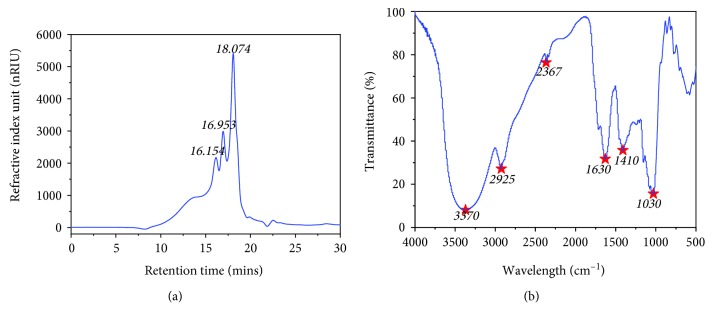
Characterization of LBP. (a) HPLC-GPC detection chromatogram of LBP. (b) Infrared spectra of LBP.

LBP had a carbohydrate content of 79.24% ± 1.75%, a protein content of 1.37% ± 0.11%, uronic acid content of 1.33 ± 0.16, and iodization reaction without starch (Table 2). After hydrolysis, it is found by GC-MS analysis that the monosaccharide composition of LBP consisted of mannose, glucose, and galactose in a molar ratio of 1.5 : 118 : 1. The HPLC-GPC chromatogram of LBP revealed that the molecular weight of LBP is composed of 4310 Da, 1910 Da, and less than 1000 Da, with a respective retention time of 16.15, 16.95, and 18.07 minutes (Figure 1(a), Table 3).

The FT-IR spectra of LBP demonstrate that LBP has a predominant carbohydrate composition (Figure 1(b)). Among the spectra, one peak (gently broad) approximately at 3370 cm^−1^ may be a characteristic vibration peak of the hydroxyl group. There is a peak caused by C-H vibration at about 2930 cm^−1^. A weak peak at around 2400 cm^−1^ is caused by CO_2_ in the air. 1630 cm^−1^ has a relatively sharp peak caused by carboxyl groups. A weak peak at around 1410 cm^−1^ is caused by C-H. A series of peaks in the range of 1000-1250 cm^−1^ might be the (C-O-C) glycosidic band vibrations and ring vibrations overlapped with the C-OH. There are a series of peaks in the range of 820-950 cm^−1^ that can be the small amount of *β*-configuration sugar, small amount of mannose, and *α*-D-glucose.

### 3.2. LBP Can Extend the Lifespan of *C. elegans*

**Figure 2:**
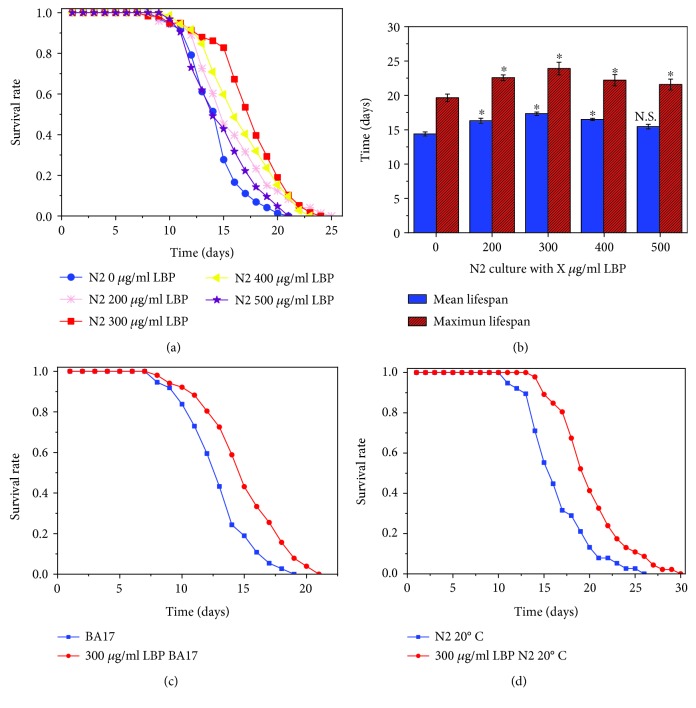
LBP can extend the lifespan of *C. elegans*. (a) At 25°C, *C. elegans* longevity was cultured at different concentrations of LBP and survival was monitored. (b) At 25°C, the mean lifespan and maximum lifespan of *C. elegans* were cultured at different concentrations of LBP. (c) At 25°C, BA17 mutant longevity was cultured with or without LBP and survival was monitored. (d) At 20°C, the survival of *C. elegans* was cultured with or without LBP. Not significant was abbreviated as N.S., ^∗^*P* < 0.05.

Lifespan is a very important indicator for measuring aging. Our study shows that for wild-type (N2) and BA17 worm strains, 300 *μ*g/ml LBP is the best concentration for lifespan extension which is temperature independent (Figures 2(a)–2(d), Table 4). At 25°C, the mean lifespan of N2 (control group) was 14.38 ± 0.30 days and the maximum lifespan was 19.64 ± 0.54 days, while the mean lifespan of N2 cultured with 300 *μ*g/ml LBP was 17.36 ± 0.24 days and the maximum lifespan was 21.99 ± 0.94 days. The results (Figures 2(a) and 2(b)) show that when feeding 300 *μ*g/ml LBP, the average lifespan expectancy and maximum lifespan of N2 are significantly extended by 20.72% and 21.69%, respectively. Under different concentrations of LBP, it can be seen that the best concentration for extending lifespan was 300 *μ*g/ml, while the extensions were not linearly related to the concentration (Figure 2(a)). BA17 worms were temperature-sensitive mutant, and they will develop into sterile adults at 25°C. Since egg production complicates survival analysis, sterile BA17 worms are also used here. Survival curves of BA17 worms cultured with or without LBP were displayed in Figure 2(c). It is shown that 300 *μ*g/ml LBP can prolong the lifespan of BA17 worms significantly (Table 4). Therefore, LBP can extend lifespan regardless whether reproductive function is minimized or not. Temperature is important for the survival of organisms; therefore, worms were also cultured with 300 *μ*g/ml LBP at 20°C (Figure 2(d), Table 4). Regardless of varying temperatures at 20°C or 25°C, cultures with 300 *μ*g/ml LBP significantly extended lifespan, so this suggests that the effect of LBP on extending lifespan is independent of temperature.

**Figure 3:**
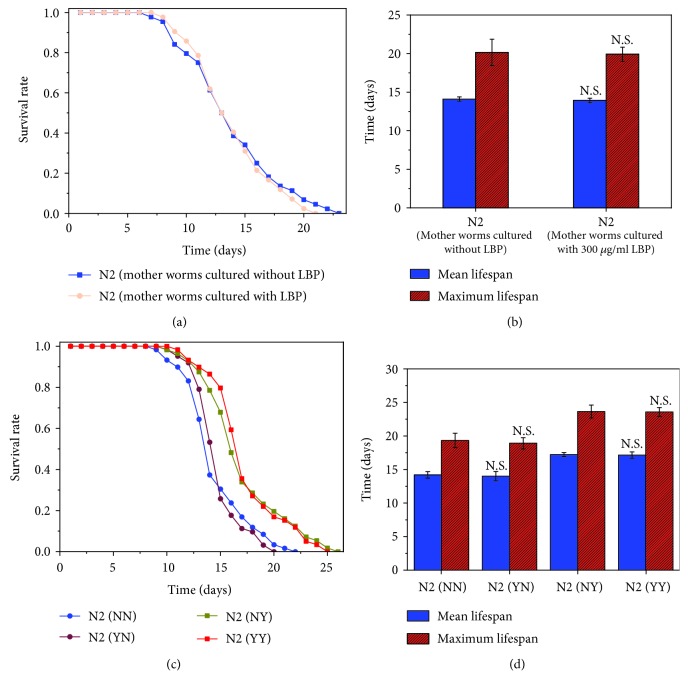
In *C. elegans*, LBP lifespan extension effects are not inheritable and do not depend on a particular stage of development. (a) Survival of offspring of N2 produced on plates with 300 *μ*g/ml LBP to standard NGM plates without LBP and the control. (b) The mean lifespan and maximum lifespan of F1 offspring of N2 produced on plates with 300 *μ*g/ml LBP to standard NGM plates without LBP and the control. (c) Survival of the worms before or after L4 as they were fed or not with LBP. (d) The mean lifespan and maximum lifespan of the worms before or after L4 as they were fed or not with LBP. Not significant was abbreviated as N.S. N2 (NN): culture without LBP; N2 (YN): culture with 300 *μ*g/ml LBP before the L4 stage but without LBP after the L4 stage; N2 (NY): culture without LBP before the L4 stage but with 300 *μ*g/ml LBP after the L4 stage; N2 (YY): culture with 300 *μ*g/ml.

The F1 offspring of N2 from plates with 300 *μ*g/ml LBP was transferred to new NGM plates without LBP, and their lifespan is compared with that of control N2. As shown in Figures 3(a) and 3(b), their lifespans were essentially the same and this implies that the effects of LBP lifespan extension cannot be inherited. In order to investigate whether LBP affects the lifespan on a particular stage of development, worms were divided into four groups (Figure 3(c), Table 4). We found that no matter whether the N2 worms were grown on plates with or without LBP before L4, there was no significant effect on the lifespan (Figures 3(c) and 3(d), Table 4).

### 3.3. LBP Can Improve the Health of *C. elegans*

**Figure 4:**
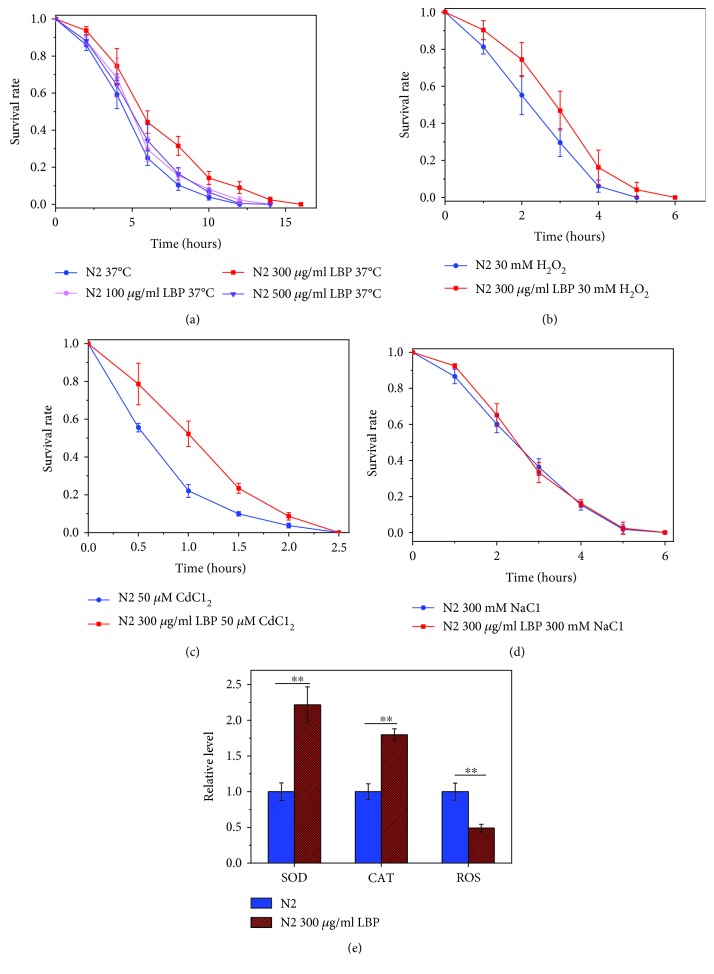
LBP improved the health of *C. elegans.* (a) Survival curves of N2 at 37°C for heat stress at different concentrations of LBP. (b) Survival of N2 cultured with or without LBP in S-buffer containing 30 mM H_2_O_2_ for oxidative stress. (c) Survival of N2 cultured with or without LBP in S-buffer containing 50 mM CdCl_2_ for metal stress. (d) Survival of N2 cultured with or without LBP in S-buffer containing 300 mM NaCl for osmotic stress. (e) The relative fluorescence values of SOD, CAT, and ROS in the unit protein concentration of worms. Not significant was abbreviated as N.S., ^∗∗^*P* < 0.01.

Animals are considered to be healthy if they have the ability to withstand environmental stresses. As can be seen from Figure 4, the lifespan of N2 cultured with 300 *μ*g/ml LBP was longer than that of the control group in heat stress, oxidative stress, and heavy metal stress, while it was almost the same as that of the control group under osmotic stress. In other words, LBP can improve the ability of *C. elegans* to resist high environmental temperature, enhance antioxidation responses, and boost heavy metal stress resistance, but with no impact on osmotic stress. We also measured the ROS, SOD, and CAT levels in the worm, and the consistent results suggested that LBP can enhance antioxidation responses of *C. elegans*. ROS is the most important factor causing oxidative damage, so the lower the total ROS level in the body, the stronger the antioxidant activity. As the main antioxidant in the body, the enzyme SOD mainly removes excess superoxide radicals from the body. CAT acts as a major peroxidase to promote the breakdown of H_2_O_2_ into molecular oxygen and water and get rid of hydrogen peroxide from the body, thereby protecting cells from H_2_O_2_ toxicity. Levels of both CAT and SOD represent the antioxidative capacities of the organism, i.e., the higher their levels, the stronger the antioxidation capacity. We found that N2 cultured with 300 *μ*g/ml LBP had lower ROS levels and higher levels of SOD and CAT (Figure 4(e)). These results were consistent with earlier results that revealed enhanced resistant to different environmental stresses.

**Figure 5:**
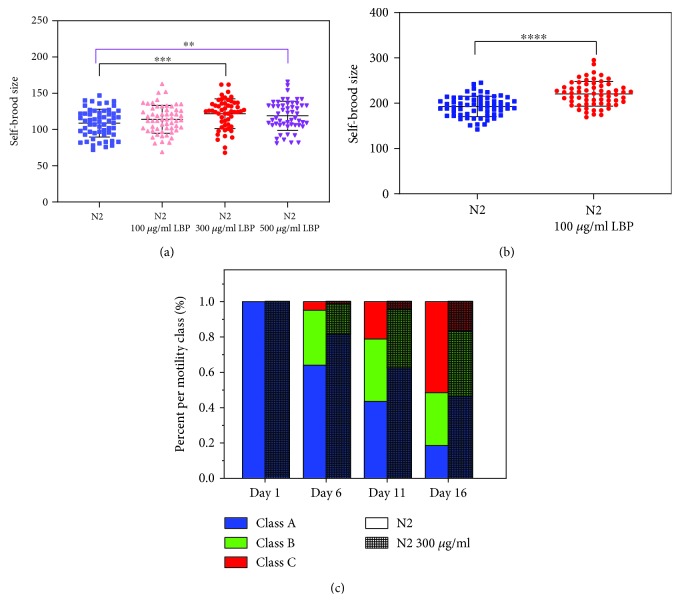
LBP enhances the worms' ability to reproduce and maintain a considerably high muscle integrity. (a) At 25°C, the progeny size and egg production rate of N2 cultured at different concentrations of LBP. (b) At 20°C, the progeny size and egg production rate of N2 cultured with or without LBP. (c) Percent of per motility class of N2 cultured with or without LBP on day 1, day 6, day 11, and day 16. ^∗∗^*P* < 0.01, ^∗∗∗^*P* < 0.001, and ^∗∗∗∗^*P* < 0.0001.

Self-brood size and egg production rate are the embodiments of reproductive ability which is a key parameter to quantify health. We evaluated the progeny size and egg production rate of the worms at 25°C and 20°C. We found that N2 cultured with 300 *μ*g/ml LBP had a higher egg production rate than those of the control group at 25°C and 20°C (Figures 5(a) and 5(b)). At 25°C, the self-brood size was 121.81 ± 20.40 against 108.77 ± 19.17 for the control group, while at 20°C, the self-brood size was 220.65 ± 27.11 against 192.98 ± 22.43 for the control group, reflecting an improvement by 11.99% and 14.34%, respectively. Motility is a measure of muscle integrity, and its measurement has a direct implication on quality living. We found that N2 cultured with 300 *μ*g/ml LBP were able to maintain a better motile state than the control group (Figure 5(c)). At day 16, there were still 46.5% of N2 cultured with 300 *μ*g/ml LBP that belong to Class A but only 18.6% of the control group was still in Class A. All these results indicate that LBP can enhance the worms' ability to reproduce and maintain considerably high muscle integrity.

### 3.4. LBP Lifespan Extension Requires daf-16 and sir-2.1

**Figure 6:**
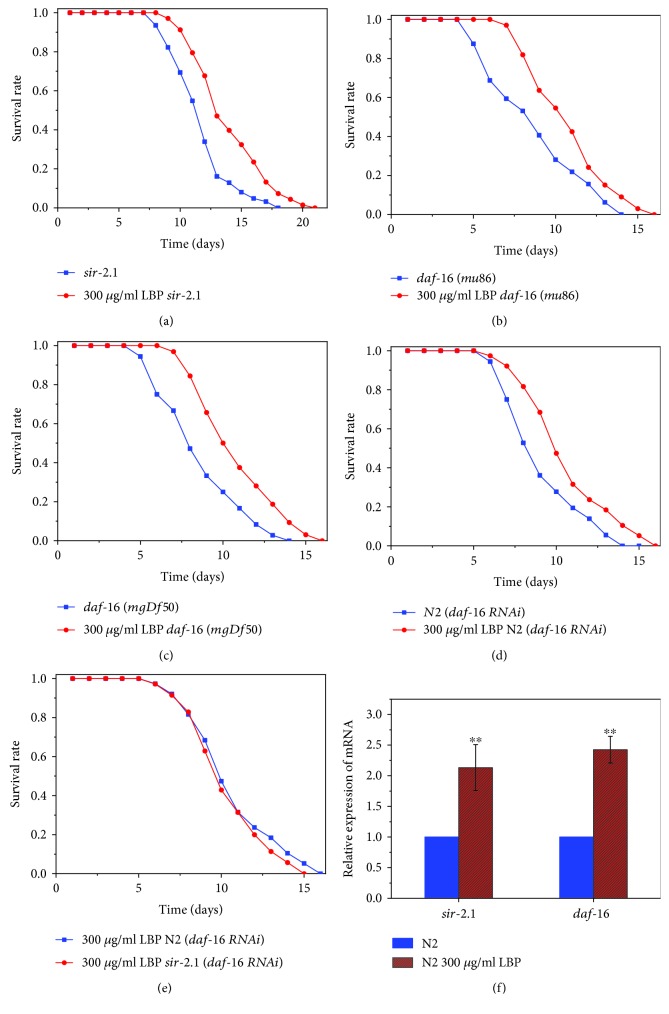
LBP lifespan extension requires *daf-16* and *sir-2.1.* (a) Survival of *sir-2.1* mutants cultured with or without LBP. (b) Survival of *daf-16* (*mu86*) mutants cultured with or without LBP. (c) Survival of *daf-16* (*mgDf50*) mutants cultured with or without LBP. (d) Survival of N2 (*daf-16* RNAi) cultured with or without LBP. (e) Survival of N2 (*daf-16* RNAi) and *sir-2.1* (*daf-16* RNAi) cultured with LBP. (f) The relative expression of *sir-2.1* and *daf-16* in N2 cultured with LBP to that of N2 cultured without LBP. ^∗∗^*P* < 0.01.

The insulin/IGF-1 signaling (IIS) pathway is undoubtedly a key regulator of longevity in a variety of animals, where the genes *daf-16* and *sir-2.1* play cardinal roles [30]. Our study showed that VC199 (ok434) mutants for *sir-2.1* deletion exhibited a significantly longer lifespan when cultured with 300 *μ*g/ml LBP but the proportion of prolonged lifespan was significantly shorter than that of the wild-type N2 (Figure 6(a), Table 4). At 25°C, the mean and maximum lifespans of worms on NGM with 25 *μ*M FUdR were 11.64 ± 0.41 days and 16.78 ± 0.35 days, respectively, for the VC199 mutants. However, when cultured with 300 *μ*g/ml, the mean and maximum lifespans were 13.18 ± 0.92 days and 18.21 ± 0.46 days, respectively. It implies that the mean and maximum lifespans were extended by 13.23% and 8.52%, respectively. LBP cannot cause such a tremendous effect on the proportion of extended lifespan in VC199 mutants compared with the N2, so LBP-induced lifespan extension requires *sir-2.1*. By RT-PCR in Figure 6(f), we obtained a *sir-2.1* fold expression of 2.43 ± 0.22 of N2 cultured with 300 *μ*g/ml LBP relative to the control group, which further proves this result.

Similarly, at 25°C, we cultured two different worm mutants, in which *daf-16* has been deleted, GR1307 (Df50) and CF1038 (mu86) (Figures 6(b) and 6(c)). We found that the lifespan of these strains cultured with 300 *μ*g/ml LBP was longer than that of the control group, but still, the proportion of the extended lifespan was significantly shorter than that of the N2, therefore implying that *daf-16* is vital in LBP lifespan extension. We also obtained similar results through *daf-16* RNAi gene silencing experiment as shown in Figure 6(d). To further confirm the importance of daf-16 expression, we performed RT-PCR of daf-16 (Figure 6(f)). We found that 300 *μ*g/ml LBP treatment significantly upregulates this gene in C. elegans, confirming that the lifespan-extending effect of LBP is mediated by the DAF-16 pathway.

In 2006, Berdichevsky et al. [31] reported that *sir-2.1* can activate *daf-16* to prolong the lifespan, which means *sir-2.1* plays a role dependent on *daf-16*. The *daf-16* RNAi experiments were performed on N2 and VC199 worm strains. We found that the results of the *daf-16* RNAi experiment were basically the same for the N2 and VC199 worm strains in cultures with 300 *μ*g/ml LBP. In the case of *daf-16* RNAi, LBP does not promote longevity of N2 or VC199 worm strains (Figure 6(e)). This suggests that the LBP's ability to extend the lifespan is through *sir-2.1* whose effect can be dependent of *daf-16*.

### 3.5. LBP Lifespan Extension Requires daf-12

**Figure 7:**
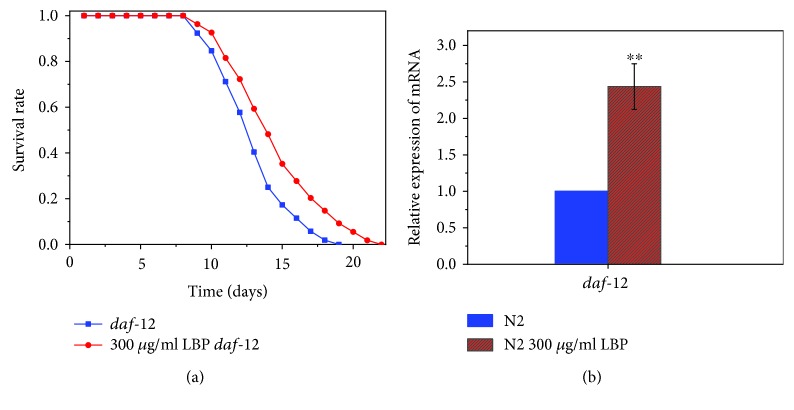
LBP lifespan extension requires *daf-12.* (a) Survival of *daf-12* mutants cultured with or without LBP. (b) The relative expression of *daf-12* in N2 cultured with LBP to that of N2 cultured without LBP. ^∗∗^*P* < 0.01.

The *daf-12* gene plays an important role in regulating somatic and reproductive development, fat metabolism, and prolonging survival [32]. The function of *daf-12* in the process of extending lifespan relies on *daf-16* or acts independently [32]. Our study shows that DR1407 (m583) mutants for *daf-12* deletion cultured with 300 *μ*g/ml LBP lived significantly longer but the proportion of extended lifespan was remarkably shorter than that of N2 (Figure 7(a), Table 4). At 25°C, the mean and maximum lifespans of DR1407 mutants were 12.54 ± 0.51 days and 18.05 ± 0.54 days, respectively. When cultured with 300 *μ*g/ml LBP, the mean and maximum lifespans of DR1407 were 13.86 ± 0.78 days and 20.82 ± 0.50 days, respectively, thereby exhibiting a 10.53% increase and a 15.35% increase, respectively, in lifespan. The role of LBP in prolonging the lifespan of worms of DR1407 strain is not as significant as that in N2, which indicates that the LBF lifespan extension effect requires *daf-12*. RT-PCR results in Figure 7(b) show that there was an increase in the *daf-12* mRNA level in the 300 *μ*g/ml LBP-treated N2 worms when compared to the control, which further proves the formidable role of *daf-12* in LBP lifespan extension.

### 3.6. LBP Extend Lifespan by daf-12/daf-16

**Figure 8:**
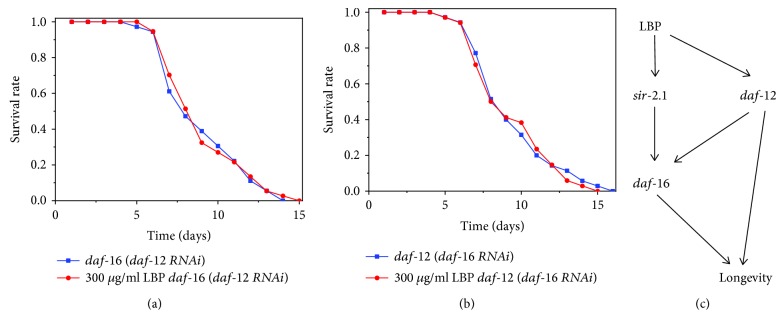
LBP lifespan extension requires *daf-16* and *daf-12.* (a) Survival of *daf-16* (*daf-12* RNAi) mutants cultured with or without LBP. (b) Survival of *daf-12* (*daf-16* RNAi) mutants cultured with or without LBP. (c) Pathway of LBP lifespan extension.

The effects of LBP on extended lifespan require *daf-16* and *daf-12*. To understand the relationship between *daf-16* and *daf-12* in the extended lifespan by LBP, we performed the RNAi treatment to silence *daf-16* in the DR1407 mutants and *daf-12* in the GR1307 mutants. We found that strains cultured with 300 *μ*g/ml LBP had almost the same lifespan as the control and there was no significant difference in lifespan between the RNAi groups (Figure 8). LBP cannot have impact on the lifespan of worms in which *daf-12* and *daf-16* were not expressed; therefore, LBP extends lifespan by *daf-12*/*daf-16*. The corresponding relationship among daf-12/daf-16 and other genes with lifespan is shown in Figure 8(c).

## 4. Discussion and Conclusions

Aging is a process regulated by many factors and is characterized by a gradual impairment of the body's response to stress and general deterioration of the main metabolic pathways [33]. Studies in various animal models, including worms, mice, and monkeys, have found that many factors, such as limiting food intake, slowing mitochondrial respiration, reducing germ cell function, or lowering temperature, can extend lifespan [34–39]. As the global aging population increases, it is one of the current research hotspots to find drugs or foods that can safely and effectively delay aging [40, 41]. We extracted a polysaccharide with a relatively stable composition from the Ningxia Lycium barbarum. LBP mainly consists of carbohydrates, most of which being glucose obtained after hydrolysis. We used *C. elegans* to examine the function of LBP in extending lifespan and maintaining health.

We studied the lifespan of *C. elegans* cultured at different concentrations of LBP and found that LBP can extend lifespan. The extended lifespan was not simply linearly dependent on the dose of LBP, and it was very interesting that there was an optimal concentration of 300 *μ*g/ml. The lifespan extension effect of LBP on worms was independent of temperature and nonheritable. LBP was also found to enhance the viability of N2 under high temperature, strong oxidation, and heavy metals. LBP promotes the antioxidant capacity of the worms, significantly increases the activity of CAT and SOD to scavenge oxidative free radicals, and suppresses the detrimental effect of ROS, thereby reducing oxidative damage of cells and improving the health. LBP can also enhance the reproductive potentials and muscle integrity of *C. elegans*. In a word, LBP did not only extend the lifespan but also improved the health status of the nematodes.

Reproduction of C. elegans was also significantly increased when worms were cultured with 300 *μ*g/ml LBP. This result seems somewhat contradictory to previous studies, which suggest that there is a tradeoff between longevity and reproduction. It has been found that long-lived C. elegans (caused by genetic mutations or nutritional disturbances) can reduce egg production. For example, reduced fertility of mutant animals, such as *age-1*, *eat-2*, and *daf-2*, is accompanied by the extension of the lifespan [42–44]. Hsin and Kenyon showed that the lifespan of germlineless C. elegans will be 60% longer than that of the wild-type animals [45]. The tradeoff relationship between longevity and reproduction may result from the energy competition between these two processes. Energy and resources used for one process come at the cost of another. However, there may be other tradeoff relationships for longevity, such as the tradeoff between lifespan extension and the decline of the pharyngeal pumping rate [46] when worms were cultured with blueberry polyphenols. In addition, recent works by Sang-Kyu Park's group [47, 48] show that N-acetyl-L-cysteine and extracts from Tenebrio molitor can extend the lifespan and increase the fertility of C. elegans at the same time. Therefore, it seems reasonable that LBP extend C. elegans lifespan without reducing fertility. There can be other tradeoffs that need further examination in future studies.

Many genetic pathways have been shown to be conserved, and it is necessary to elucidate the molecular mechanisms by which LBP extends lifespan [49–52]. *sir-2.1* and *daf-16* are extremely outstanding genes in the IIS pathway; as well, DAF-16/FOXO transcription factors are also key components of the IIS pathway associating with aging and metabolism [30, 31]. We found that LBP has a shorter extending lifespan effect on mutants of *sir-2.1* and *daf-16* than on N2. This means that the LBP-extended lifespan effect requires *sir-2.1* and *daf-16*. For *daf-16* RNAi, there is no difference in the effect of LBP on N2 and *sir-2.1* mutants, which implies that *sir-2.1* may play a role dependent on *daf-16*. The *daf-12* gene plays an important role in aging, immunity, and antioxidation [32]. We found that LBP had a shorter lifespan extension effect on *daf-12* mutants than on N2, implying that the LBP-extended lifespan requires *daf-12*. *daf-16* RNAi was done to the *daf-12* mutants, and *daf-12* RNAi was done to the *daf-16* mutants, and it was shown that LBP lost the function of prolonging the lifespan, which suggests the effects of LBP on the aging of *C. elegans* depend on *daf-12* and *daf-16*.

In summary, a polysaccharide was obtained from Lycium barbarum, which has the functions of prolonging the lifespan and maintaining the environmental adaptability, reproductive capacity, and motility of *C. elegans*. The effects of LBP on the health and aging of *C. elegans* are regulated by *sir-2.1*, *daf-12*, and *daf-16*. Our study presented the preparation, basic chemical properties, and composition of LBP; its role in aging and health; and its molecular mechanism. It also provided a basis for further research on LBP and LBP as a food or drug for intervention of aging.

## Figures and Tables

**Table 1:** qPCR primer sequence.

Primer name		Primer sequence
act-1	F	5′-CTACGAACTTCCTGACGGACAAG-3′
	R	5′-CCGGCGGACTCCATACC-3′

sir-2.1	F	5′-GCAACGATTCAGAAGTTGCG-3′
	R	5′-TGTGCATAGAACCGATTTCTGG-3′

daf-16	F	5′-ATCGTGTGCTCAGAATCC-3′
	R	5′-ATGAATAGCTGCCCTCC-3′

daf-12	F	5′-AAACGAAGAACAACTGCGGC-3′
	R	5′-TGTGGTGACTGCTGATTCCC-3′

**Table 2:** Chemical compositions of LBP.

No.	Compositions	Percentage of quality (%)
1	Carbohydrate	79.24 ± 1.75
2	Uronic acid	1.33 ± 0.16
3	Protein content	1.37 ± 0.11
4	Starch	(-)

**Table 3:** Mw of main component peaks of LBP.

No.	Retention times (mins)	Mw (Da)
1	16.15	4.31*E*+03
2	16.95	1.91*E*+03
3	18.07	<1000

**Table 4:** Summary of all lifespan experiments shown in this work.

No.	Strains	Bacteria	LBP (*μ*g/ml)	Temperature (°C)	Mean lifespan (days)	Max. lifespan (days)	Compared with no.	*P* (log-rank test)
1	N2	*E. coli* OP50	0	25	14.38 ± 0.30	19.64 ± 0.54	/	/
2	N2	*E. coli* OP50	50	25	15.30 ± 0.30	20.98 ± 0.29	1	0.1393
3	N2	*E. coli* OP50	100	25	15.84 ± 0.39	21.99 ± 0.94	1	0.0368
4	N2	*E. coli* OP50	200	25	16.31 ± 0.37	22.57 ± 0.41	1	0.0013
5	N2	*E. coli* OP50	300	25	17.34 ± 0.24	23.90 ± 0.92	1	<0.0001
6	N2	*E. coli* OP50	400	25	16.50 ± 0.16	22.20 ± 0.81	1	<0.0001
7	N2	*E. coli* OP50	500	25	15.44 ± 0.34	21.57 ± 0.79	1	0.1592
8	BA17	*E. coli* OP50	0	25	12.89 ± 0.36	17.73 ± 0.48	/	/
9	BA17	*E. coli* OP50	300	25	15.18 ± 0.23	19.82 ± 0.41	8	0.0006
10	N2	*E. coli* OP50	0	20	16.98 ± 0.52	24.60 ± 0.73	/	/
11	N2	*E. coli* OP50	300	20	19.83 ± 0.35	27.33 ± 0.94	10	<0.0001
12	N2	*E. coli* OP50	0 (mother worms: 0)	25	14.10 ± 0.27	20.15 ± 0.72	/	/
13	N2	*E. coli* OP50	0 (mother worms: 300)	25	13.92 ± 0.30	19.92 ± 0.92	12	0.6266
14	N2	*E. coli* OP50	0	25	14.21 ± 0.47	19.33 ± 1.09	/	/
15	N2	*E. coli* OP50	L1-L4: 300 L4: 0	25	14.02 ± 0.70	18.91 ± 0.84	14	0.9505
16	N2	*E. coli* OP50	L1-L4: 0 L4: 300	25	17.22 ± 0.30	23.64 ± 0.94	14	<0.0001
17	N2	*E. coli* OP50	300	25	17.15 ± 0.47	23.57 ± 0.66	16	0.8412
18	*sir-2.1*	*E. coli* OP50	0	25	11.64 ± 0.41	16.78 ± 0.35	/	/
19	*sir-2.1*	*E. coli* OP50	300	25	13.18 ± 0.92	18.21 ± 0.46	18	<0.0001
20	*daf-16* (mu86)	*E. coli* OP50	0	25	9.79 ± 0.85	14.11 ± 0.51	/	/
21	*daf-16* (mu86)	*E. coli* OP50	300	25	10.98 ± 0.08	15.33 ± 0.67	20	0.0126
22	*daf-16* (Df50)	*E. coli* OP50	0	25	9.45 ± 0.66	13.67 ± 0.34	/	/
23	*daf-16* (Df50)	*E. coli* OP50	300	25	11.16 ± 0.66	15.80 ± 0.50	22	0.001
24	N2	*daf-16* RNAi	0	25	9.78 ± 0.51	14.95 ± 0.63	/	/
25	N2	*daf-16* RNAi	300	25	10.99 ± 0.21	16.11 ± 0.51	24	0.0095
26	*sir-2.1*	*daf-16* RNAi	300	25	10.89 ± 0.66	15.81 ± 1.17	25	0.4269
27	*daf-12*	*E. coli* OP50	0	25	12.54 ± 0.51	18.05 ± 0.54	/	/
28	*daf-12*	*E. coli* OP50	300	25	13.86 ± 0.78	20.82 ± 0.50	27	0.0033
29	*daf-16*	*daf-12* RNAi	0	25	9.47 ± 0.46	13.64 ± 0.13	/	/
30	*daf-16*	*daf-12* RNAi	300	25	9.73 ± 0.40	14.75 ± 1.54	29	0.7915
31	*daf-12*	*daf-16* RNAi	0	25	9.37 ± 0.66	13.56 ± 0.51	/	/
32	*daf-12*	*daf-16* RNAi	300	25	9.40 ± 0.47	13.92 ± 0.74	31	0.1554

## Data Availability

The data used to support the findings of this study are available from the corresponding author upon request.
